# 立体定向放疗后原发病灶旁新发快速进展磨玻璃型肺腺癌1例

**DOI:** 10.3779/j.issn.1009-3419.2023.106.25

**Published:** 2023-12-20

**Authors:** Sicong WANG, Linfeng LI, Yuanda CHENG

**Affiliations:** 410008 长沙，中南大学湘雅医院胸外科; Department of Thoracic Surgery, Xiangya Hospital, Central South University, Changsha 410008, China

**Keywords:** 肺肿瘤, 立体定向放疗, 磨玻璃结节, 进展, Lung neoplasms, Stereotactic body radiation therapy, Ground-glass nodule, Progression

## Abstract

磨玻璃型肺癌临床常表现为惰性，其接受立体定向放疗（stereotactic body radiation therapy, SBRT）治疗后长期随访的临床研究较少。本文提供1例接受SBRT治疗的磨玻璃型肺癌的成功案例，但随访中发现临近SBRT治疗靶区的肺组织内，新发1例磨玻璃结节。该结节进展迅速，经手术切除证实为肺腺癌，但分子病理结果及基因检测未见明显高危因素和相关驱动基因。新发磨玻璃结节是否与既往SBRT治疗有关，值得进一步研究。本案例提示，SBRT治疗后的随访复查，应警惕靶区及临近肺组织内新发快速进展型肺癌的发生。

## Abstract (en)

Ground-glass nodule (GGN) lung cancer often progresses slowly in clinical and there are few clinical studies on long-term follow-up of patients with operable GGN lung cancer treated with stereotactic body radiation therapy (SBRT). We present a successful case of GGN lung cancer treated with SBRT, but a new GGN was found in the lung adjacent to the SBRT target during follow-up. The nodule progressed rapidly and was confirmed as lung adenocarcinoma by surgical resection. No significant risk factors and related driving genes were found in molecular pathological findings and genetic tests. It deserves further study whether new GGN is related to the SBRT. This case suggests that the follow-up after SBRT should be vigilant against the occurrence of new rapidly progressive lung cancer in the target area and adjacent lung tissue.

## 1 病例资料

患者，男，49岁，因“左上肺腺癌行立体定向放疗（stereotactic body radiation therapy, SBRT）治疗后4年9个月，发现左上肺磨玻璃结节（ground-glass nodule, GGN）3年余”在中南大学湘雅医院胸外科再次就诊。患者于2018年10月体检发现左上肺尖后段混合GGN，大小约1.8 cm×1.3 cm（图1A），计算机断层扫描（computed tomography, CT）引导下肺结节穿刺活检，病理诊断为“腺癌”。患者拒绝手术，要求行SBRT治疗，于2018年12月10日至12月19日期间行SBRT治疗（剂量为60 Gy/8 f），后多次CT复查提示病灶逐渐缩小并呈纤维化条索状改变（图1B-1F）。2020年5月25日CT复查提示左上肺尖后段新发GGN，大小约3 mm，临近原SBRT治疗靶区，2023年4月17日CT示结节较前明显增大，呈高密度GGN改变，大小约为9 mm（图2）。患者于2023年6月26日接受胸腔镜下左上肺叶切除+淋巴结清扫（4/5/7/10/11组），术后病理结果：腺癌，贴壁型为主，神经、脉管均未见侵犯；基因检测结果：未见I/II类基因突变；原SBRT治疗病灶部位呈纤维组织增生，未见癌细胞；免疫组化：甲状腺转录因子1（thyroid transcription factor-1, TTF-1）（+），天冬氨酸蛋白酶A（novel aspartic proteinase A, Napsin A）（+），Ki67（6%+），P53（野生型），癌胚抗原（carcinoembryonic antigen, CEA）（-），程序性死亡配体1（programmed cell death ligand 1, PD-L1）（E1L3N）（肿瘤比例评分：<1%）。 患者术后顺利出院，目前随访恢复良好。该病例报道已获患者及家属知情同意。

**图1:**
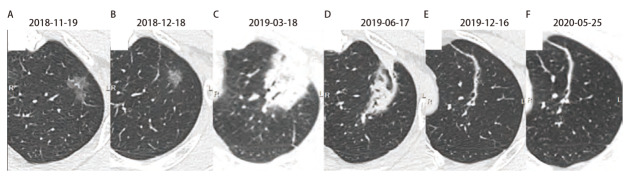
左上肺尖后段结节SBRT治疗前后及随访CT影像改变。A：SBRT治疗前原发病灶，左上肺尖段GGN改变，大小约为1.8 cm×1.3 cm；B-F：SBRT治疗后，不同时间段CT影像学改变，病变先缩小，局部呈放射性肺炎改变，然后逐渐呈纤维条索状改变。

**图2:**
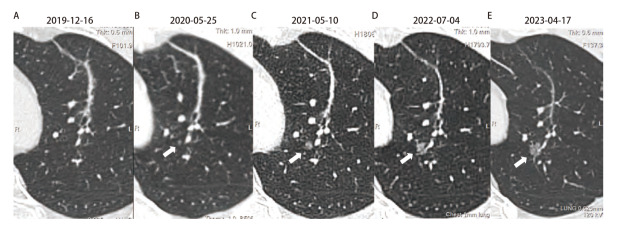
左上肺原SBRT治疗靶区附近新发GGN及随访CT影像变化。A：2019年12月16日胸部CT复查原SBRT治疗区域呈条索状改变，临近区域未见新发结节；B-E：2020年5月25日胸CT复查，原SBRT治疗区域临近区域新发微小结节（白色箭头处），大小约3 mm，呈GGN改变，后定期复查，该结节逐渐增大，2021年5月10日复查大小约6 mm，2022年7月4日复查大小约7 mm，2023年4月17日复查大小约9 mm。

## 2 讨论

肺癌发病率及死亡率在我国均高居第一位，外科手术是早期非小细胞肺癌（non-small cell lung cancer, NSCLC）的首选治疗方式^[1]^，然而，对于拒绝手术治疗或者身体无法耐受手术治疗的患者，SBRT或射频消融（radiofrequency ablation, RFA）是可选择的局部治疗手段。多篇文献^[2⇓-4]^指出，对于可手术的早期NSCLC患者，SBRT的总体生存率低于手术治疗，但癌症特异性生存率无统计学差异。同时，SBRT对于原发肿瘤的控制率在80%以上，且对于身体基础情况不佳、难以耐受手术的患者，严重毒性的概率也相当低^[5,6]^。目前，SBRT可作为拒绝手术或无法耐受手术治疗的早期NSCLC患者的首选治疗方法。本例患者左上肺原发病灶经过SBRT治疗后，效果良好，近5年随访未见明显局部复发迹象，且术后病理证实原发病灶呈纤维化改变，未见癌细胞，达到病理上的治愈状态。

但在本案例中，左上肺新发GGN值得关注。对于稳定增长的亚实性结节而言，纯GGN（pure GGN, pGGN）的进展较缓慢。一项回顾性分析^[7]^指出，pGGN中位体积倍增时间为769 d，在增大的pGGN中，91.7%的患者pGGN体积倍增时间超过400 d。临床中大多数GGN进展缓慢，表现出“惰性”，但在本案例中，患者新发现的pGGN进展较快，表现出浸润性快速生长的趋势（图2），且利用Schwartz公式^[8]^，我们计算出本例中GGN体积倍增时间约为116.7 d，这与临床上发现的大部分GGN不同。基于该结节进展较快，与原SBRT治疗靶区较近，不排除肺内转移可能，手术选择了肺叶切除，并进行了淋巴结清扫。患者术后病理结果显示，神经、脉管未见侵犯，Ki67表达并不高（6%），PD-L1表达阴性，淋巴结未见转移，基因检测未见有临床意义的癌症相关驱动基因突变。因此，本例中的GGN进展迅速的原因不清。Kim等^[9]^的研究表明，肺腺癌的实性成分占比与经气道播散（spread through air spaces, STAS）具有独立相关性，而在pGGN组中无STAS现象。本例中患者新发GGN，无明显实性成分，且病理结果显示无淋巴结转移和STAS。综上，该患者新发GGN型肺癌无目前已知的分子病理上的高危因素，其快速生长的原因值得深入研究。研究^[10,11]^表明，SBRT可在治疗区域内调节肿瘤微环境，诱导肿瘤细胞产生新突变和新的融合基因，进而产生新的特异性抗原，提高肿瘤免疫原性；但有文献^[12]^同时指出，高剂量的射线同时会带来副反应，如原发部位淋巴细胞减少、局部和循环中髓系来源的抑制细胞以及调节性T细胞增加，从而抑制肿瘤对免疫治疗的反应。不同剂量的SBRT治疗对组织内免疫微环境产生了不同的影响，不同的免疫微环境也为新发肿瘤的发生提供了可能。本案例中，新发GGN型肺腺癌与原SBRT治疗的病灶距离较近，毗邻SBRT治疗的靶区范围，其发生和发展是否与SBRT治疗带来的射线辐射致癌相关，也需要大样本的临床研究进一步分析。

综上，从本案例可见，SBRT治疗早期NSCLC能够达到病理上长期治愈的可能，而对于SBRT治疗后的追踪复查，应警惕靶区及临近的肺组织新发快速进展型肺癌的发生。

**Conflicts of interest**

The authors declare that they have no competing interests.
